# Supplementary material for: Acute febrile illness among outpatients seeking health care in Bangladeshi hospitals prior to the COVID-19 pandemic
Source: PLoS One. 2022 Sep 1;17(9):e0273902. doi: 10.1371/journal.pone.0273902 (PMC9436081; doi:10.1371/journal.pone.0273902)
Supplement: S1 Table — (DOCX) [file pone.0273902.s001.docx]

**S1 Table. List of pathogens and tests used for AFI surveillance, Bangladesh**

| **Test** | **Where conducted** | **Pathogens** | **Specific assay** |
| --- | --- | --- | --- |
| Rapid diagnostic test (RDT) | Hospitals | Dengue | NS1 |
|  |  | Chikungunya | IgM and IgG |
|  |  | Zika | IgM and IgG |
|  |  | Malaria | pf/pv |
| PCR* | icddr,b | *Rickettsia spp.* |  |
|  |  | *Orientia tsutsugamushi* |  |
|  |  | *Brucella spp.* |  |
|  |  | *C. Burnetii* |  |
|  |  | Crimean–Congo hemorrhagic fever (CCHF) |  |
|  |  | Hepatitis E |  |
|  |  | *Leptospira spp.* |  |
|  |  | Zika |  |
|  |  | West Nile |  |
|  |  |  |  |
| Blood and urine culture | icddr,b | Aerobic bacteria |  |
|  |  |  |  |
| PCR* | U.S. CDC | *Rickettsia spp.* |  |
| Serology | U.S. CDC | *Rickettsia spp.* |  |
| Microscopic agglutination test (MAT) | U.S. CDC | *Leptospira spp.* |  |

**PCR= Polymerase chain reaction*
